# Letter to the Editor regarding “Social media use and roles of self-objectification, self-compassion and body image concerns: a systematic review”

**DOI:** 10.1186/s40337-026-01751-2

**Published:** 2026-08-31

**Authors:** Dwi Prasetyo, Tia Saraswati

**Affiliations:** 1 Department of Communication Science, Universitas Negeri Surabaya, Surabaya, Indonesia; 2 Department of Japanese Literature, Universitas Airlangga, Surabaya, Indonesia

**Keywords:** Social media use, Self-objectification, Self-compassion, Body image concerns, Communication psychology, Social media literacy, Eating disorders

## Abstract

The systematic review by Sarda et al. synthesizes relationships among social media use, self-objectification, self-compassion, and body image concerns while evaluating evidence relevant to the revised objectification theory model proposed by Wollast and colleagues. This letter extends the authors’ call for more rigorous examination of social media use by arguing for greater specificity in conceptualizing digitally mediated appearance experiences. Rather than treating social media as a single exposure or relying on a broad passive/active distinction, we propose examining configurations of content, communicative practices, interpersonal feedback, social comparison, and platform-level curation as “patterned communicative experiences.” These experiences can be investigated through activity-specific measures, ecological momentary assessment, digital traces, content analysis, and longitudinal designs. Self-compassion should remain an intrapersonal psychological construct, while communication environments are better treated as contextual conditions that may facilitate or inhibit its activation. Integrating objectification theory, communication psychology, and social media literacy may therefore clarify when and how digital experiences contribute to body surveillance, body shame, and eating disorder risk.

**Dear Editor,**

We read with great interest the systematic review by Sarda et al. [1], which synthesizes research on social media use, self-objectification, self-compassion, and body image concerns. The review evaluates evidence in relation to the revised objectification theory model proposed by Wollast et al. [2], in which self-compassion is positioned as a potential protective factor against self-objectification. This distinction matters: the revised model originates from Wollast and colleagues, while Sarda et al. assess the extent to which empirical findings support relationships specified within that framework. Their review provides a foundation for more precise accounts of how interactive digital environments may shape body-related self-perception and eating disorder risk.

We would like to extend one future direction already identified by Sarda et al. [1]: the need for more rigorous examination of different dimensions of social media use and exposure. Studies in the review operationalized social media through Facebook intensity, appearance-related conversations and comparisons, abstinence, and appearance-related cyberbullying. The next methodological step should go beyond replacing “screen time” with another global indicator by operationalizing social media use as a patterned communicative experience composed of interacting dimensions.

At least four dimensions appear theoretically useful: (1) content, including appearance-ideal, sexualized, body-positive, body-neutral, or non-appearance-related material; (2) communicative practices, such as posting photographs, editing selfies, searching for appearance information, discussing bodies, comparing appearances, or responding to others’ content; (3) relational and evaluative cues, including comments, peer responses, influencer messages, cyberbullying, and visible engagement metrics; and (4) platform-level curation, including repeated recommendations and the prominence of appearance-related material within algorithmically organized feeds. These dimensions can be examined separately and in combination rather than collapsed into a single measure of “social media use.”

Within this framework, symbolic validation refers to socially visible signals that a particular self-presentation is recognized or valued by others, such as likes, positive appearance-related comments, shares, follower responses, or comparable engagement indicators. Although these cues may differ in meaning across users or platforms, repeated attachment to appearance-focused self-presentation may create an environment in which appearance is both socially evaluated and quantitatively visible. This offers a communication mechanism through which the external gaze central to objectification theory may remain salient in digital settings.

Operationalizing such experiences is more demanding than measuring duration or frequency, but feasible approaches are available. Future studies could combine short-window, activity-specific self-report with ecological momentary assessment, asking participants after selected social media sessions what content they encountered, what they did, whether comparison occurred, what feedback they received, and whether body surveillance or dissatisfaction changed. Where feasible, digital trace data could document posting and interaction patterns, while content coding could estimate the density and type of appearance-related material encountered. Longitudinal and multilevel models could distinguish between-person from within-person changes, while experiments could manipulate visible versus hidden engagement metrics, appearance-focused versus body-neutral feedback, or curated content streams.

We also suggest caution regarding the broad distinction between “passive” and “active” social media use. Two active behaviors for example, privately messaging a supportive friend and repeatedly editing and posting an appearance-focused selfie may have very different implications for self-objectification. Likewise, viewing content may involve different processes depending on whether users encounter friends, influencers, appearance ideals, body-neutral material, or algorithmically recommended posts. The theoretically relevant distinction may lie less in active versus passive behavior than in communicative function, content, comparison processes, and social feedback. This mechanism-specific approach is consistent with calls to move beyond treating social media as a uniform exposure [3].

Such specificity may be particularly important for understanding self-objectification. Self-objectification may arise not only through exposure to idealized bodies but also through repeated participation in environments structured around appearance evaluation. Likes, appearance-focused comments, filters, comparison practices, and repeated algorithmic presentation of idealized imagery can make an external evaluative perspective salient. The body may consequently become something repeatedly monitored, compared, modified, presented, and evaluated. Experimental and longitudinal evidence links appearance-focused social media exposure with body image outcomes [4]; systematic reviews document adverse effects of exposure to beauty ideals [5]; and meta-analytic evidence indicates associations between online social comparison, body image concerns, and eating disorder symptoms [6]. A communication-centered operationalization may therefore help specify mechanisms connecting digital experiences with body surveillance and body shame within objectification theory.

We would also refine the interpretation of self-compassion. It should remain conceptualized as an intrapersonal psychological resource involving self-kindness, common humanity, and mindful rather than judgmental responses to personal difficulty. We do not propose a collective or system-level form of self-compassion. Instead, communication environments may constitute contextual conditions under which individual self-compassion is more or less likely to be activated, sustained, or challenged. Supportive responses following appearance-related distress may facilitate self-kindness, whereas appearance criticism, body talk, cyberbullying, or comparison-oriented interaction may increase self-critical responses.

Community-level characteristics could be operationalized independently through perceived social support, body-accepting norms, appearance-related peer norms, or supportive communication climate, while self-compassion remains measured at the individual level. Research could test whether self-compassion buffers associations between appearance-evaluative communication and body shame, and whether supportive communication environments strengthen that buffering effect. Longitudinal and ecological momentary designs could also examine reciprocal dynamics: whether digital experiences predict temporary changes in self-compassion and whether people with higher self-compassion subsequently engage differently with comparison, feedback seeking, or self-presentation. Such designs provide a stronger basis for directionality than cross-sectional associations alone.

These distinctions also matter for prevention. Sarda et al. [1] highlight self-compassion and media literacy as promising intervention targets. Rather than focusing only on reducing exposure, interventions could help users recognize the communicative mechanisms through which appearance pressure is produced: comparison triggers, visual framing and editing, repeated recommendations, dependence on appearance-contingent feedback, and interaction norms. Brief self-compassion interventions have shown potential for protecting body image in appearance-related social media contexts [7]. Their effectiveness may be strengthened by social media literacy approaches that help users understand how platforms organize attention, visibility, persuasion, and comparison [8].

In conclusion, Sarda et al. [1] provide an important foundation for examining self-compassion in research on social media, self-objectification, and body image concerns and for evaluating the revised objectification model proposed by Wollast et al. [2]. An important next step is methodological specificity. Rather than asking only how much social media people use or reducing engagement to a passive/active distinction, future studies could examine combinations of content, communicative practices, interpersonal evaluation, comparison processes, and platform-level curation. This approach does not require redefining self-compassion as a collective construct; instead, it allows researchers to examine how an individual psychological resource operates within communication environments that may intensify or reduce appearance-based evaluation. Combining specified communication mechanisms with longitudinal, experimental, ecological momentary, content-analytic, and digital-trace approaches may help identify not simply whether social media is associated with body image concerns, but which digitally mediated experiences create risk, for whom, through what mechanisms, and under what conditions self-compassion may be protective.

## Data Availability

No datasets were generated or analysed during the current study.

## Declarations

### Declarations of generative AI and AI-assisted technologies

We declare that Grammarly is used to assist with grammatical checking. QuillBot is also employed to support sentence paraphrasing and enhance linguistic nuance. However, all AI-generated suggestions are carefully reviewed by the authors to ensure accuracy.
